# Seed characterization and early nitrogen metabolism performance of seedlings from Altiplano and coastal ecotypes of Quinoa

**DOI:** 10.1186/s12870-020-02542-w

**Published:** 2020-07-21

**Authors:** Katherine Pinto-Irish, Teodoro Coba de la Peña, Enrique Ostria-Gallardo, Cristian Ibáñez, Vilbett Briones, Alexander Vergara, Rodrigo Alvarez, Catalina Castro, Carolina Sanhueza, Patricio A. Castro, Luisa Bascuñán-Godoy

**Affiliations:** 1 Centro de Estudios Avanzados en Zonas Áridas (CEAZA), 1710088 La Serena, Chile; 2 Departamento de Biología, Universidad de La Serena, Av. Raúl Bitrán 1305, 1710088 La Serena, Chile; 3 Departamento de Ingeniería en Alimentos, Universidad de La Serena, La Serena, Chile; 4 Umeå Plant Science Centre, Department of Forest Genetics and Plant Physiology, Swedish University of Agricultural Sciences, SE901 83, Umeå, Sweden; 5 Escuela de Tecnología Médica, Facultad de Salud, Sede La Serena, Universidad Santo Tomás, La Serena, 1710172, Chile; 6 Laboratorio de Fisiología Vegetal, Departamento de Botánica, Facultad de Ciencias Naturales y Oceanográficas, Universidad de Concepción, Casilla 160-C, 4070386 Concepción, Chile; 7 Departamento de Fisiología, Facultad de Ciencias Biológicas, Universidad de Concepción, 4070386 Concepción, Chile

**Keywords:** Nitrogen metabolism, N transport, Glutamine synthetase, Photochemical process, Chlorophyll fluorescence, Nitrate reductase, Nitrate transporters, Nitrate-sensing, Proteins, Crop yield

## Abstract

**Background:**

Early seed germination and a functional root system development during establishment are crucial attributes contributing to nutrient competence under marginal nutrient soil conditions. *Chenopodium quinoa* Willd (Chenopodiaceae) is a rustic crop, able to grow in marginal areas. Altiplano and Coastal/Lowlands are two representative zones of quinoa cultivation in South America with contrasting soil fertility and edaphoclimatic conditions.

In the present work, we hypothesize that the ecotypes of Quinoa from Altiplano (landrace Socaire) and from Coastal/Lowland (landrace Faro) have developed differential adaptive responses in order to survive under conditions of low availability of N in their respective climatic zones of Altiplano and Lowlands. In order to understand intrinsic differences for N competence between landraces, seed metabolite profile and germinative capacity were studied. Additionally, in order to elucidate the mechanisms of N uptake and assimilation at limiting N conditions during establishment, germinated seeds of both landraces were grown at either sufficient nitrate (HN) or low nitrate (LN) supply. We studied the photosynthetic performance, protein storage, root morphometrical parameters, activity and expression of N-assimilating enzymes, and the expression of nitrate transporters of roots in plants submitted to the different treatments.

**Results:**

Seeds from Socaire landrace presented higher content of free N-related metabolites and faster seed germination rate compared to Faro landrace. Seedlings of both ecotypes presented similar physiological performance at HN supply, but at LN supply their differences were exalted. At LN, Socaire plants showed an increased root biomass (including a higher number and total length of lateral roots), a differential regulation of a nitrate transporter (a *NPF6.3*-like homologue) belonging to the Low Affinity Transport System (LATS), and an upregulation of a nitrate transporter (a *NRT2.1*-like homologue) belonging to the High Affinity nitrate Transport System (HATS) compared to Faro. These responses as a whole could be linked to a higher amount of stored proteins in leaves, associated to an enhanced photochemical performance in Altiplano plants, in comparison to Lowland quinoa plants.

**Conclusions:**

These differential characteristics of Socaire over Faro plants could involve an adaptation to enhanced nitrate uptake under the brutal unfavorable climate conditions of Altiplano.

## Background

Nitrogen is a nutrient of foremost importance to predict physiological performance and yield of plants [1]. Nitrogen (N) is a component of many macromolecules including nucleic acids and proteins. Plant N compounds also include components of the ornithine cycle and the shikimate pathway [2].

Nitrogen stored in seeds has an important ecological role because it is involved in seed predation, protection and germination [3, 4]. Furthermore, it was suggested that metabolites stored in seeds represent a defined metabolic status and they may play a regulatory role over gene expression, defining levels of dormancy or/and ripening [5, 6]. Additionally, metabolites in seeds play a major role in early performance of species under poor nutrient conditions [7, 8].

After germination, the ability of seedlings for N uptake and assimilation from soils depends on various factors including, available form of N, presence of other nutrients, soil characteristics, pH, temperature, abiotic factors, biotic interactions, competition, root structure, etc. In most plant species, only a small proportion of soil N is taken up and assimilated by plant roots. Plant can capture different inorganic forms of N in the roots, but nitrate (NO_3_^−^) is the major N source absorbed and assimilated by plants in aerobic soils [9]. Nitrate reductase (NR) catalyzes the first reaction in nitrate assimilation, the reduction of nitrate to nitrite. Nitrite reduction to ammonium is catalyzed by the enzyme nitrite reductase (NiR). Then, glutamine synthetase (GS) incorporates ammonium into glutamine in the GS-GOGAT cycle, converting inorganic N into organic N in plants [10].

Plants actively take up NO_3_^−^ from soil thanks to the activity of root specific transporters via a proton/nitrate-coupled mechanism [11]. NO_3_^−^ uptake transporters are classified in two categories: Low Affinity Transport System (LATS) and High affinity Transport System (HATS) [12]. It was observed in Arabidopsis that LATS is mainly involved in transport at high nitrate concentrations, while HATS is active at low nitrate conditions (< 1 mM) [13, 14].

*Chenopodium quinoa* (Chenopodiaceae) seeds have a high protein content and a complete amino acid composition. Additionally, because of its wide adaptability and ability to grow under unfavorable environmental conditions, quinoa has been declared of global importance for food sustainability by the Food and Agricultural Organization [15].

Quinoa was domesticated approximately in 3000 B.C., probably on the high plateaus of the Altiplano of Peru and Bolivia [16]. Quinoa was spread out by the Inca Empire along the whole Andean mountain range, from Colombia to Southern Chile [17]. Today, there are more than 6000 landraces of Quinoa cultivated by farmers worldwide.

The Altiplano and Lowlands are representative geographical areas where Quinoa plants are submitted to contrasting environmental conditions in Chile. The Altiplano is immersed in the Atacama Desert in northern Chile. Quinoa plants growing in the Altiplano are exposed to extreme stressful climatic conditions called “cold desert”. These conditions include high-flux solar radiation, very strong diurnal changes of temperature, limited volume of annual rainfall (150–300 mm/year) and saline soils [18]. On the other hand, quinoa Lowland ecotypes (from central and southern Chile) have to deal with acidic and impoverished soils, elevated temperatures during growth and rainfall ranges from 500 to 1500 mm/year [19]. Both places and their edaphoclimatic conditions are representative of the wide stressful conditions (that reduce the probability of nutrient uptake and assimilation) Quinoa must cope with in Chile.

We hypothesize that ecotypes of Quinoa from Altiplano (landrace Socaire) and from Lowland (landrace Faro) have developed differential adaptive responses in order to survive under conditions of low availability of N in the different climatic zones. In this regard, we wanted to elucidate whether the contrasting landraces of Quinoa store different level of N metabolites on seeds that could determine a differential speed of germination and competitiveness for nutrients. Thus, we determined the N-related metabolite composition of seeds and their germinative capacity. Further, seedlings of both landraces were grown at either sufficient nitrate (HN) or low nitrate (LN) supply for 20 days, and several parameters were determined in order to elucidate the mechanisms of N uptake and assimilation at limiting N conditions during establishment of each quinoa landrace. These parameters included morphometrical measures, photosynthetic performance, protein storage, as well as activities and gene expression of N-assimilating enzymes (NR, GS). A phylogenetic analysis of quinoa LATS and HATS homologues was performed, and relative quantification of expression of a selected LATS gene (a *NPF6.3*-like homologue) and a selected HATS gene (a *NRT2.1*-like homologue) were also performed. We found evidence for a differential performance regarding N uptake between landraces.

## Results

### Seed germination, nitrogen content and metabolomic profiling

**Fig. 1:**
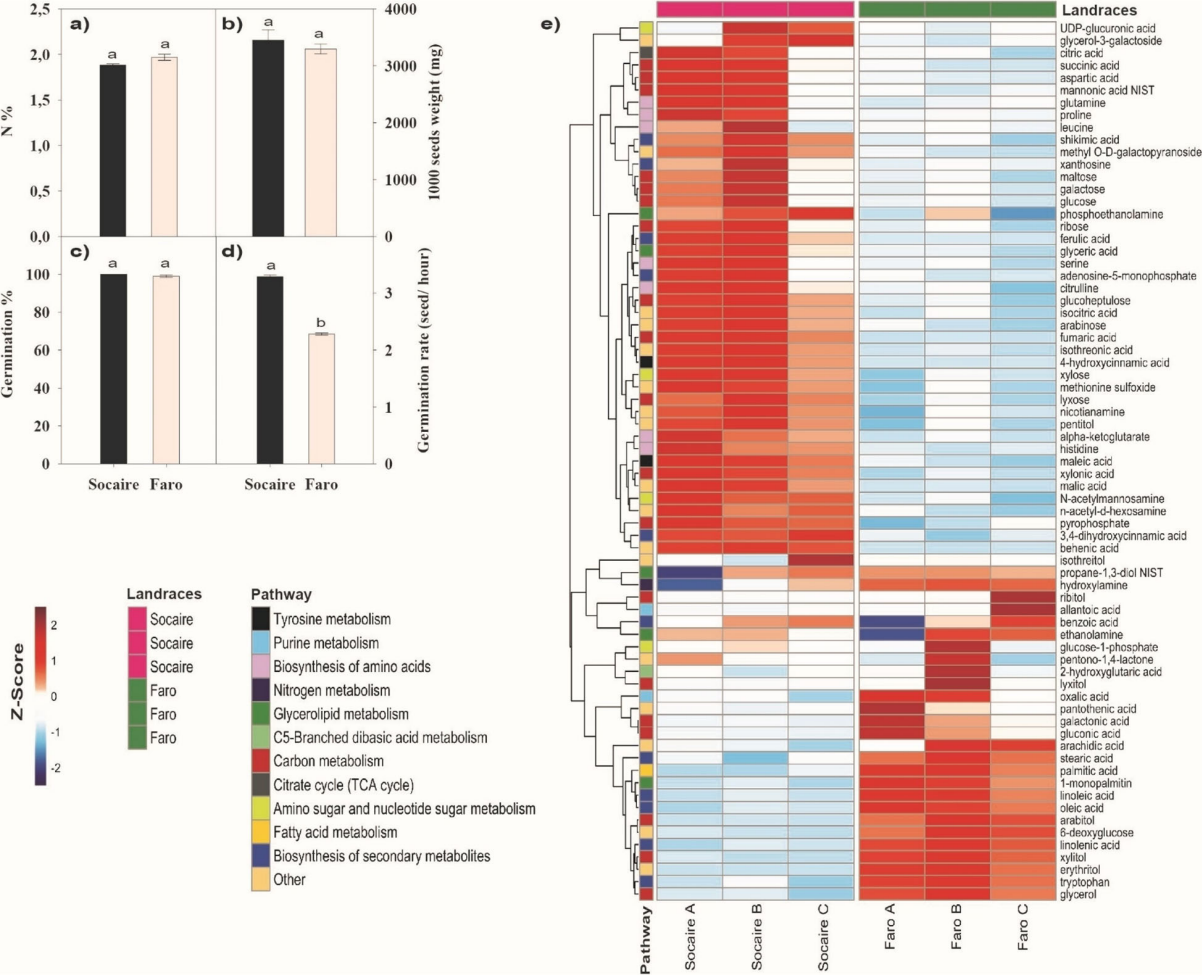
Seed-related parameters in two landraces (Socaire and Faro) of Quinoa. (**a**) seed N content, (**b**) weight of 1000 seeds (**c**) percentage of germination and **d**) germination rate were determined in seeds of each landrace. Bars show mean values ± SE (*n* = 5). Different letters represent significant differences among landraces and treatments at *P <* 0.05 using two-way ANOVA. **e**) Heat map of selected metabolites with pathway information from KEGG. Only metabolites with low variance between replicates are showed. Normalized abundances of metabolites were scaled by Z-score and represented by a color scale. Letters after landrace (**a, b, c**) indicate different samples (50 mg) from three different plants (*n* = 3). Original full data set of all metabolites abundances are shown in Suppl. Table S1

Both Socaire and Faro had similar seed weights and N content (Fig. 1a and b). Even though both landraces reached 100% germination after 48 h of imbibition (Fig. 1c). Socaire seeds displayed a significanty higher germination rate than that observed in Faro (3.5 versus 2.3 germinated seeds/h; Fig. 1d). Socaire reached an earlier total germination at 16 h, versus 24 h in the case of Faro seeds (Suppl. Fig. S1).

A seed metabolite profile, comparing Socaire and Faro landraces, was performed. A total of 161 named metabolites were confirmed using an untargeted global metabolomic platform that combined Gas Chromatography - Mass spectrometry (GC - MS) and Ultra performance Liquid Chromatography - Tandem Mass Spectrometer (UPLC/ MS/ MS) analyses of seeds from both landraces. The results revealed significant differences in metabolite content between landraces, which reflect the variations in seed origin and/or genetic background (Fig. 1e).

**Fig. 2:**
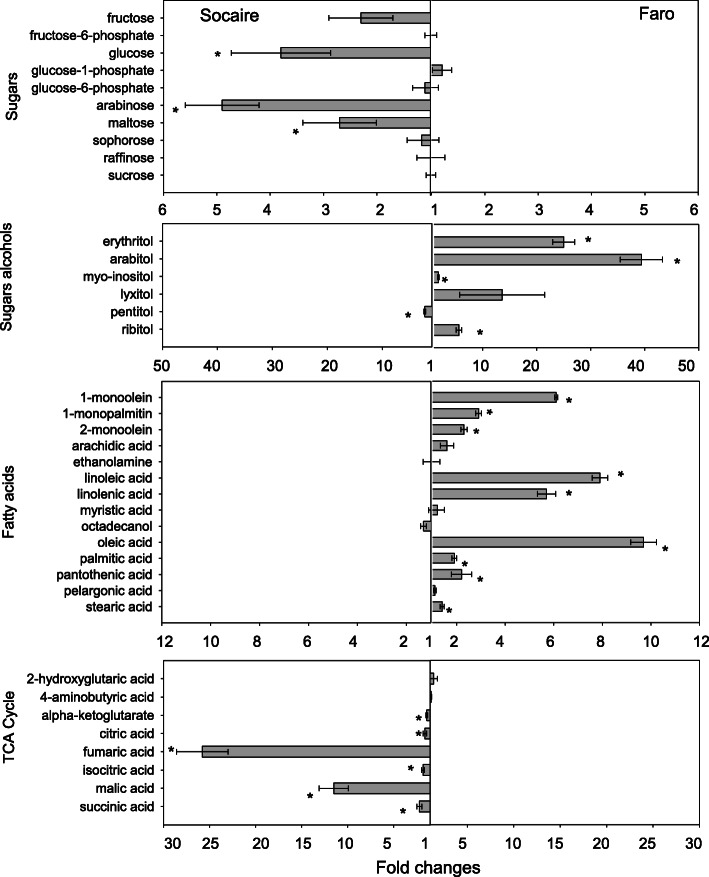
C-related metabolites in seeds of two landraces of *C. quinoa*. Value bars facing the left of each section indicate the relative fold changes content of Socaire seed compared to Faro. Value bars facing the right of each section indicate relative increased content in Faro seeds compared to Socaire. Values are representative of mean ± SE of three independent pool of seeds. Asterisks show significant differences between treatments or landraces using one-way ANOVA followed by Fisher LSD test; *P* < 0.05

**Fig. 3:**
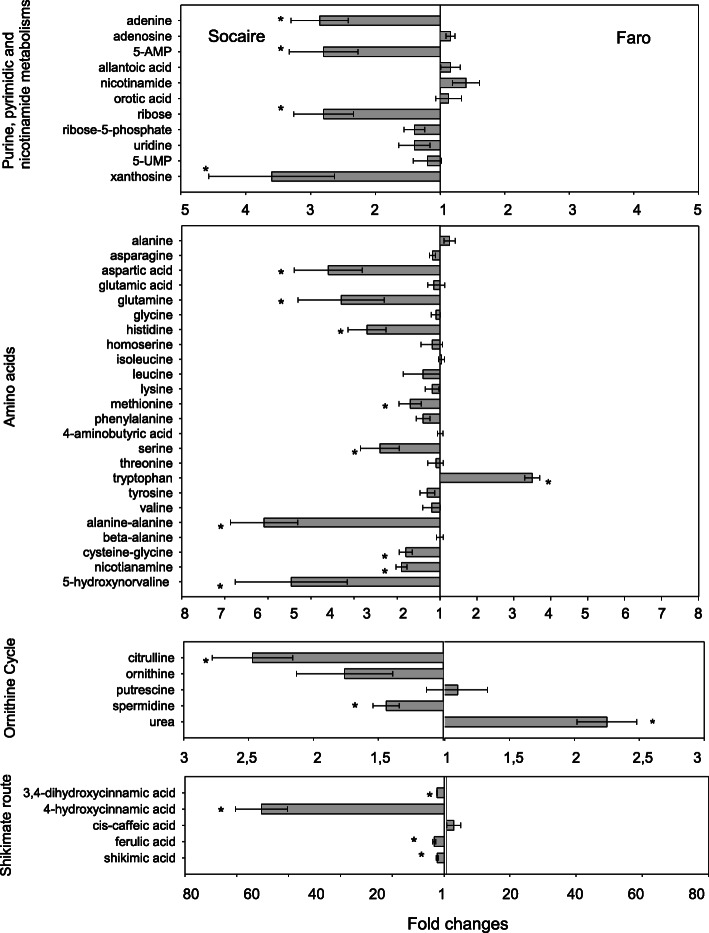
N-related metabolites in seeds of two *C. quinoa* landraces. Value bars facing the left of each section indicate the relative fold changes in content of these metabolites in Socaire seeds compared to Faro. Value bars facing the right of each section indicate relative increased content in Faro seeds compared to Socaire. Values are representative of mean ± SE of three independent pool of seeds. Asterisks show significant differences between treatments or landraces using one-way ANOVA followed by Fisher LSD test; *P* < 0.05

The confirmed 161 metabolites covered most of the primary metabolism and some of the secondary metabolism pathways. Metabolites were categorized into eight major metabolite groups including C- and N-related pathways: sugars, sugars alcohols, fatty acids, TCA cycle, free aminoacids, purine and pirimidin metabolism, ornithine cycle and shikimic acid route (Figs. 1, 2 and 3e).

Similar levels of the most significant fraction of water-soluble carbohydrates and glycolytic intermediates including sucrose, raffinose, glucose-6-phosphate, glucose-1-phosphate fructose-6-phosphate, 3-phosphoglyceraldehyde, phosphoenolpyruvate and pyruvate were found when comparing both landraces (Figs. 2 and 3). However, Socaire seeds had higher amounts of glucose, fructose and other more complex sugars (such as lyxose, arabinose, galactose and maltose) in comparison to Faro seeds.

Faro seeds had higher contents of sugar acids, sugar alcohols and ketose sugars, including galactonic acid (sugar acid), mio-inositol, ribitol, lyxitol, arabitol and erythritol. In addition, Faro seeds presented higher levels of fatty acids (including stearic acid, pelargonic acid, pantothenic acid, palmitic acid, oleic acid, octadecanol, myristic acid, linolenic acid, linoleic acid, arachidic acid, 1-monoolein, 2-monoolein and 1-monopalmitin), in comparison to Socaire seeds (Fig. 2). Regarding metabolites involved in the TCA cycle, Socaire seeds had higher amounts of all organic acids detected (citric acid, fumaric acid, isocitric acid, maleic acid, malic acid and alpha-ketoglutarate) (Fig. 2).

Socaire seeds also had higher levels of free N-related metabolites (non-associated to macromolecules) in comparison to Faro seeds (Fig. 3). Socaire seeds presented higher amounts of metabolites related to the purine- and pyrimidine pathway (including: xanthosine, adenine, adenosine-5-monophosphate and ribose-5P; Fig. 3). Aditionally, significantly higher amounts of amino acids (aspartic acid, glutamine, histidine, proline and serine), including non-proteinic amino acids (hydroxynorvaline and nicotianamine), and dipeptides (cysteine-glycine and alanine-alanine) were found in Socaire seeds. The only exception was tryptophan, which was present in higher amounts in Faro than in Socaire seeds.

Regarding the ornithine pathway, Socaire seeds presented higher amounts of ornithine, citruline and the polyamine spermidine (Fig. 3). Concerning the shikimate pathway, Socaire seeds had higher amounts of shikimic acid, p-cumaric acid (4-hydroxycinnamic acid), caffeic acid (3,4-dihydroxycinnamic acid) and ferulic acid than Faro seeds.

### Effect of nitrate supply on growth and physiological parameters of Socaire and Faro seedlings

Aditionally to the seed study in both landraces, we evaluated the performance of plants under different NO_3_^−^ supply conditions. To determine sufficient and limiting NO_3_^−^ concentrations, we studied growth of both plant ecoytpes at different N concentrations. It was observed that NO_3_^−^ supply had significant effects on plant growth (Suppl. Fig. S2a). Shoot fresh weight and leaf area increased with increased NO_3_^−^ supply, up to reach maximum values when growing in presence of 20 mM NO_3_^−^ and decreased at 100 mM (Suppl. Fig. S2). Leaf area and shoot weight were significantly lower, between 0 and 5 mM NO_3_^−^. Plant irrigation with 0.5 mM NO_3_^−^ resulted in 80% less shoot weight and leaf area in both landraces compared to 20 mM NO_3_^−^. Therefore, 20 mM and 0.5 mM NO_3_^−^ were chosen as high nitrogen (HN) and low nitrogen (LN) supply treatment for subsequent experiments, respectively.

**Fig. 4:**
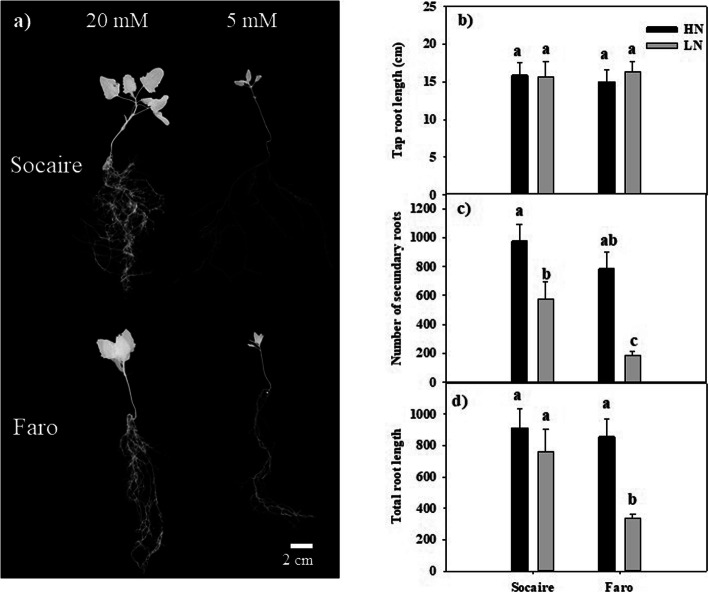
The effect of N deficiency on root morphology in seedlings of *C. quinoa***.** Images of plants subjected to HN (20 mM NO_3_^−^) and LN (0.5 mM of NO_3_^−^) supplies for 24 days. (**a**) Tips root length, (**b**) Primary root length and (**c**) number of secondary roots. Values are mean ± SE (*n* = 7). Different letters show statistical differences using two-way ANOVA considering landraces and nutrition treatment (HN and LN) as factors (Fisher LSD test; *P* < 0.05)

Seedlings of both landraces were grown for 24 days at HN or LN conditions. Although no significant differences in root length between both quinoa landraces in presence of HN and LN supply were observed (Fig. 4), the number of secondary roots and total root length were significantly higher in Socaire plants than in Faro plants at LN supply (Fig. 4c and d). Socaire plants displayed a significantly higher root/shoot ratio than Faro plants at LN supply (Table 1).

**Table 1:** Measures of leaf area and dry biomass in *C. quinoa* plants grown at two NO_3_^−^ levels. Leaf area, root dry weight, shoot dry weight, shoot/root ratio and total biomass of plants grown at sufficient nitrate supply (HN, 20 mM NO_3_^−^) and low nitrate supply (LN, 0.5 mM of NO_3_^−^) for 24 days. Values are means ± SE (*n* = 7). Different letters show statistical differences using two-way ANOVA considering landraces and nitrate treatment (HN and LN) as factors (Fisher LSD test; *P ≤* 0.05)

	Socaire		Faro	
	HN	LN	HN	LN
Leaf area (cm^2^)	3.5 ± 0.30 (b)	0.6 ± 0.06 (c)	4.0 ± 0.22 (a)	0.5 ± 0.03 (c)
Root weight (mg)	17.9 ± 2.8 (a)	2.8 ± 0.2 (b)	17.0 ± 1.2 (a)	2.4 ± 0.2 (b)
Shoot weight (mg)	37.2 ± 7.0 (a)	6.2 ± 0.8 (b)	47.6 ± 6.2 (a)	8.3 ± 0.6 (b)
Shoot/root ratio	2.4 ± 0.6 (ab)	2.2 ± 0.2 (b)	2.9 ± 0.4 (ab)	3.4 ± 0.2 (a)
Total biomass (mg)	55.1 ± 6.0 (a)	9.0 ± 0.9 (b)	64.6 ± 6.4 (a)	10.8 ± 0.6 (b)

**Fig. 5:**
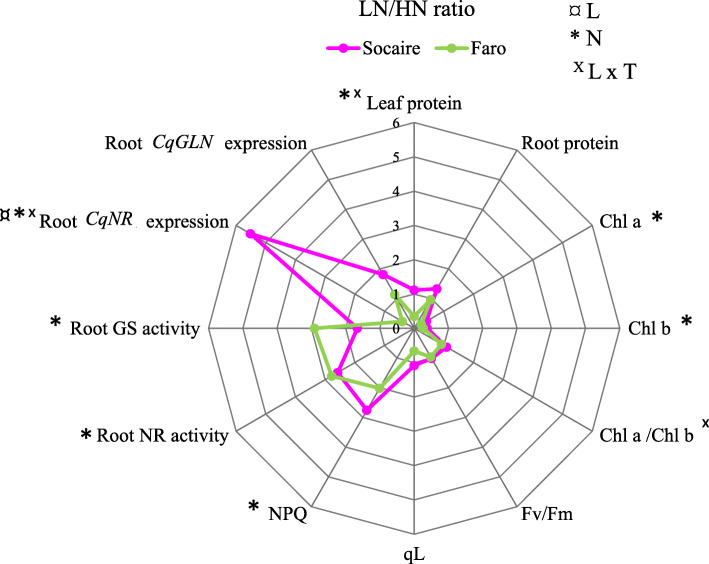
Physiological parameter changes in response to LN in Socaire and Faro. Radar chart shows relative changes in leaf and root proteins, chlorophylls (Chl *a* and *b* and Chl *a*/*b* ratio), chlorophyll fluorescence parameters Fv/Fm (maximal efficiency of PSII), NPQ (non-photochemical quenching) and qL (the proportion QA in the reduced state), protein and NR and GS enzymatic activities and their respective gene expressions (*CqNR* and *CqGLN*) in two landraces of quinoa. Changes were calculated as the ratio of LN level (0.5 mM of NO_3_^−^) to HN level (20 mM NO_3_^−^). Values are means (*n* = 5). Socaire ratio changes were denoted in pink color and Faro in green. Symbols ^**¤**^, * and ^X^ indicate significant differences among landraces (L), nitrogen treatments (N) and interaction among both factors (L x N) using two-way ANOVA (Fisher LSD test; *P <* 0.05). Data for statistical analysis are shown in Suppl. Table S2

Comparative leaf and root protein concentration, chlorophyll content and fluorescence, nitrate reductase (NR) and glutamine synthetase (GS) enzymatic activities, and their expression levels (*CqNR* and *CqGLN*) were determined in both quinoa landraces growing at HN and LN supply. Changes in these parameters were calculated as the ratio of each value at low nitrogen supply (LN) to its value at HN conditions (LN vs HN) for both landraces in a radar chart (Fig. 5).

At LN supply, a significant decrease in leaf protein concentration was observed in Faro but not in Socaire plants (Fig. 5). Nitrogen supply had not significant effect on root protein concentration in any of both quinoa landraces (Fig. 5).

Both quinoa landraces showed a decrease of about 70% in chlorophylls content when growing at LN supply (Fig. 5); thus, the Chl*a*/Chl*b* ratio is maintained in spite of N supply treatment. No differences in this response were found among landraces.

Fluorescence parameters of chlorophyll *a* were quantified in order to understand the effect of N supply on the photosynthetic apparatus. Fv/Fm is an indicator of stress in plants, and it represents the maximum quantum efficiency of Photosystem II. No significant differences in Fv/Fm values were found when comparing both landraces, and nitrate supply had not a significant effect on this parameter (Fig. 5).

The photochemical quenching (qL) reflects the proportion of energy used on photochemical process, whilst the non-photochemical quenching (NPQ) is an indicator of photoprotective mechanisms and its enhancement is related to thermal dissipation of excess of absorbed light energy. We found that, at LN conditions, both landraces were able to disipate the excess of energy as heat, but a decrease of 30% in the value of qL was observed in Faro plants, whilst no significant decrease was observed in Socaire plants (Fig. 5).

Nitrogen supply had a significant effect on NR and GS activities in both Socaire and Faro roots. Activities of both enzymes were significantly higher in LN than in HN conditions (Fig. 5). This response was concomitant with an upregulation (of about 6-fold) of the *CqNR* gene in Socaire plants, but not in Faro, whose transcripts decreased to half. In the same way, only Socaire presented a significant increment on the *CqGLN1* transcript, not observed in Faro plants.

### Phylogenetic analysis and gene expression of quinoa nitrate transporter homologues

**Fig. 6:**
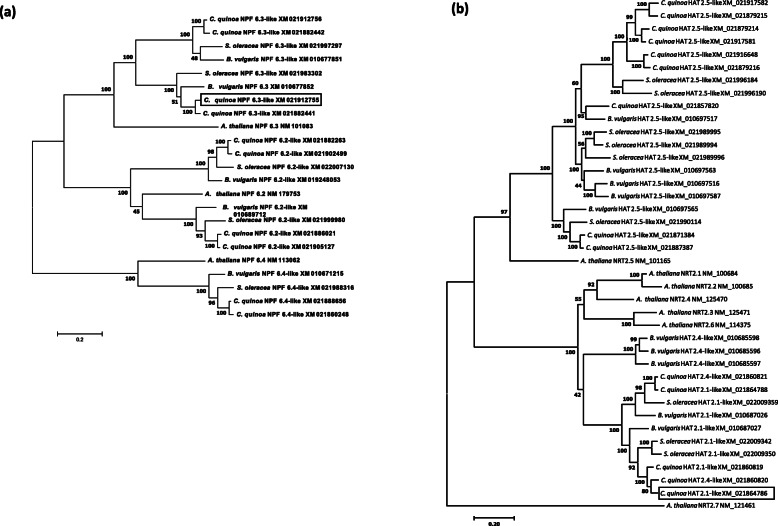
Molecular phylogenetic analyses of low and high affinity nitrate transporters in *C. quinoa*. Phylogenetic optimal trees of nucleotide coding sequences of nitrate transporter homologues of *C. quinoa*, other two species of the Chenopodiaceae family (*S. oleracea* and *B. vulgaris*), and *A. thaliana*. The evolutionary history was inferred using the Maximum-Likelihood method based on the Tamura-Nei model. In the phylogenetic analysis of Low Affinity Transporter (LAT) homologues (**a**), the tree with the highest log likelihood (− 21,210.48) is shown. In the phylogenetic analysis of high affinity transporters (HATs) (**b**), the tree with the highest log likelihood (− 29,578.42) is shown. The percentage of trees in which the associated taxa clustered together is shown next to the branches. Initial tree(s) for the heuristic search were obtained automatically by applying Neighbor-Joining and BioNJ algorithms to a matrix of pairwise distances estimated using the Maximum Composite Likelihood (MCL) approach, and then selecting the topology with superior log likelihood value. A discrete Gamma distribution was used to model evolutionary rate differences among sites. The tree is drawn to scale, with branch lengths measured in the number of substitutions per site. The analysis involved 23 (**a**) and 40 (**b**) nucleotide sequences. All positions with less than 95% site coverage were eliminated. Accession number for each sequence is indicated. Gene homologues characterized by gene expression in this study are contained in boxes. Evolutionary analyses were conducted in MEGA7

Phylogenetic analyses of some low-affinity nitrate transporters (*NRT1/PTR Family*, or *NPF*) and all high-affinity nitrate transporter (*NRT2*) homologues identified up to date in *C. quinoa* were performed (Fig. 6). In these analyses, nitrate transporter homologues from *Arabidopsis thaliana* and from two other plant species of the Chenopodioideae subfamily (*Beta vulgaris* and *Spinacia oleracea*) were also included.

Regarding LATS genes, several homologues of *C. quinoa, S. oleracea and B. vulgaris* clustered with *A. thaliana NPF 6.3* genes (Fig. 6a). Other *C. quinoa* (but neither *S. oleracea* nor *B. vulgaris*) homologues clustered with *NPF 6.2* and *NPF 6.4* genes of *A. thaliana*, forming the respective clades (Fig. 5a). In each of these clusters, *C. quinoa* presented a higher number of homologues than the other species.

Regarding high-affinity transporters (HATS), several homologues of *C. quinoa*, *S. oleracea* and *B. vulgaris* clustered together with the *A. thaliana NRT 2.5* gene (Fig. 6b). Several other homologues of the three species of the Chenopodioideae subfamily clustered with a clade containing *A. thaliana NRT 2.1*, *NRT 2.2*, *NRT 2.3*, *NRT 2.4* and *NRT 2.6* genes. No homologues to *A. thaliana NRT 2.7* gene were found (Fig. 6b).

In order to perform gene expression studies of *C. quinoa* LATS and HATS homologues, transcriptional levels of *NPF 6.3-like* (Acc. No. XM_02191275) and *HAT 2.1-like* (Acc. No. XM_021864786) gene homologues were analyzed by real-time PCR using gene-specific primers.

**Fig. 7:**
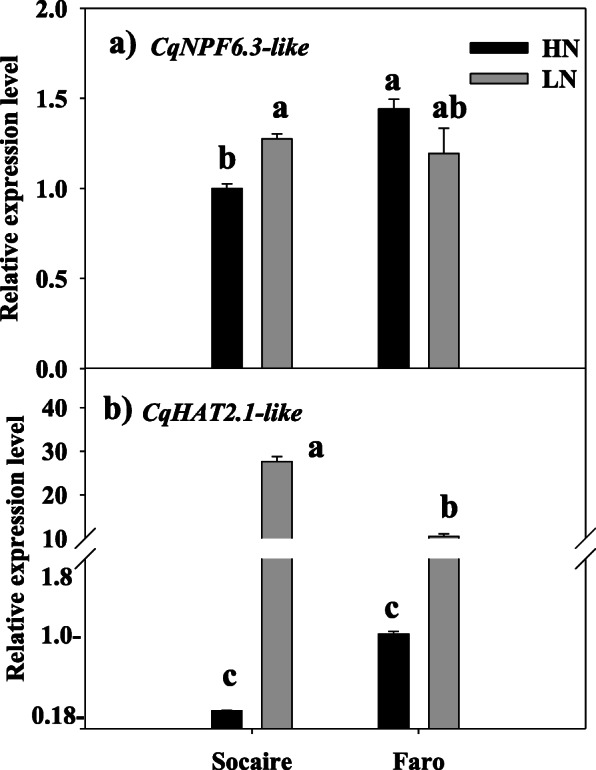
Relative expression levels of low and high affinity nitrate transporters in root of *C. quinoa* plants grown at two levels of nitrate. Relative transcript levels of low (*NPF6.3-like*) (**a**) and high (*HATS 2.1-like*, (**b**) affinity nitrate transporters in roots of two landraces of *C. quinoa* seedlings grown at HN (20 mM NO_3_^−^) and LN (0.5 mM of NO_3_^−^) for 24 days. Total RNA prepared from root tissues was analyzed by quantitative real-time PCR analysis. The constitutively expressed *EF1-α* gene was used as endogenous control in order to normalize experimental results. Mean values ± SE (*n* = 3) are represented. Different letters show statistical differences using two-way ANOVA considering landraces and nitrate supply conditions as factors (Fisher LSD test; *P* < 0.05)

Transcriptional levels of the two nitrate transporter genes were detected in roots of Socaire and Faro plants. It was observed that nitrate supply had a significant effect on expression of these low-affinity and high-affinity nitrate transporters in quinoa. A significant upregulation of the *NPF 6.3-like* homologue was observed in Socaire, but not in Faro plants, at LN supply (Fig. 7a). Additionally, a significant increase in mRNA levels of the *HAT 2.1-like* homologue was observed in both Socaire and Faro plants at LN supply, in comparison with transcript levels observed at HN supply (Fig. 7b).

## Discussion

### Seed composition of landraces

Influence of contrasting environments and genetic background on seed compounds of different quinoa landraces had been focused principally from a human nutritional perspective [20]. Notwithstanding, our metabolic seed characterization attempts to unveil the contrasting physiological performance of two landraces from the seed stage.

Typically, it was observed in ecological studies that total seed N concentration was an important factor for the release of dormancy [5, 6, 8]. Our results have shown that plants adapted to contrasting climatic conditions (Altiplano and Lowland) and local management procedures produced seed with similar weight and N content (Fig. 1), however presented differential rate of germination (Fig. 1d and Fig. S1). These results suggest that N content per se does not reflect the wide differences in N metabolites complexity, distribution, rate of use and fate during germination [8]. It was suggested that a higher free-metabolite content could favor the rate of water imbibition and could have a role as a fast fuel of energy in the early phase of germination [5, 6]. This is consistent with our results, in which Socaire, the landrace with higher storage of free metabolites also displayed a faster germination (Figs. 1, 2 and 3). Our metabolomics analysis highlighted the large metabolite differences between landraces from Altiplano and Lowland areas. The metabolites that exhibit highest range of values classified the landraces into two opposite sets, according to their level in seeds (Fig. 1e). These results are in accordance with those of Chen et al. [20], who classified the quinoa varieties into two groups regarding their bioactive phytochemicals.

It is interesting to note that Socaire seeds had higher levels of carbohydrates with higher calorific sugars, such as glucose and fructose, that can support the energetic cost for germination serving as carbon skeletons in the TCA cycle (malate, isocitric acid, succinic acid, fumarate) [21]. On the other hand, Faro seeds stores higher amounts of alcohol sugars, considered “sweetener” with low caloric power (0.4 kcal/g) such as arabitol and erythritol. Furthermore, Faro seeds store higher amount of oleic acid, linolenic acid and linoleic acid (fatty acid metabolites) than Socaire seeds. Several studies in oilseed plants demonstrated that free fatty acids and glycerol improve oxidative stability of seed through conversion into sugars, supporting TCA cycle and improving seed germination and seedling establishment processes [22].

Another noteworthy fact is the higher content of N-related metabolites in Socaire than in Faro seeds. Among the processes involved in activation of seed metabolism upon imbibition, purine salvage pathway (and not “de novo” synthesis) has been the proposed as a route for purine nucleotide synthesis, because of their favorable energetic reactions with lower ATP cost [23]. Purine pathway-related metabolites including AMP, UMP, xanthosine and aspartate (among others), were present in higher amounts in Socaire than in Faro seeds (Fig. 3). It is suggested that the higher content of purine-related metabolites, ribose 5-P and TCA intermediates observed in Socaire, could be contributing to the different enzymatic reactions related with the main pathways associated with energy production and nucleic acid biosynthesis after during seed germination [23, 24].

The aromatic amino acids tryptophan, phenylalanine and tyrosine are precursors for a wide range of secondary metabolites that are important for plant growth as well as for human nutrition and health. The occurrence of higher levels of shikimic acid, caffeic acid (3,4-dihydroxycinnamic acid), p-cumaric acid (4-hydroxycinnamic acid) and ferulic acid in Socaire seeds suggests an upregulation of secondary metabolism in this landrace in comparison with that of Faro seeds. It was reported that variations on these metabolites in *Amaranthus* seeds could be due to different climatic conditions of the provenances, but also to their genetic background [25]. Interestingly, tryptophan was the only amino acid present in higher amount in Faro than in Socaire seeds and, in agreement with our data, this amino acid is a retarder of germination in wheat [26].

Other important amino acids that were enhanced in Socaire compared to Faro seeds were glutamine and proline (Fig. 3). Both amino acids are involved in the ornithine cycle and polyamine pathway. Additionally, citrulline and spermidine levels were 2-fold higher in Socaire than in Faro seeds. All these high nitrogen content compounds were related to drought and salinity tolerance in quinoa [27, 28].

Certainly, these differential levels of metabolites in the dry seed do not necessarily imply a direct effect on germination, but many works had suggested that the levels and proportions of some metabolites in the seed affect processes required for later germination or even establishment [29].

### Differences in seedlings performance were revealed at low NO_3_^−^ supply

In order to continue with studies of developmental processes, we were interested in understanding the early performance of these quinoa plants at sufficient (HN) and low NO_3_^−^ (LN) supply conditions. Interestingly, when both landraces were grown at HN conditions, they presented similar responses in the parameters studied. However, at LN supply, differences in their physiological performances were revealed.

Both landraces produced similar total biomass at both nitrogen supply conditions 24 days after germination (Table 1), but the shoot: root ratio was significantly different between landraces at LN supply, indicating that Socaire allocated a higher proportion of resources in the root than Faro seedlings under those conditions. It is known that roots, and specifically root tips, are important sites where plant root absorbs water and nutrients [1]. Lateral roots proliferate preferentially in nutrient-rich zones and are repressed at insufficient N supply [30]. Our results showed that, similarly to other species, LN supply induced a reduction in the number of lateral roots, compared to HN conditions, in *C. quinoa*. Nevertheless, Socaire displayed a higher number of lateral roots and higher total root length than Faro seedlings at LN supply (Fig. 4). Taking into account that it was observed a higher leaf protein concentration in Socaire than in Faro seedlings (Fig. 5), we suggest that maintenance of a higher number of lateral roots might be a crucial factor contributing to the higher N accumulation observed in Socaire ecotype.

It is known that the vast majority of N is stored principally in leaves as Rubisco and other proteins from the light harvesting complex in the thylakoid system of plants [1]. Observed decreases in the levels of chlorophylls in both landraces under LN conditions (Fig. 5) could be the result of adjustments of the photosynthetic apparatus to reduce the energy captured by plants to avoid photodamage. The ability for photosynthetic apparatus adjustment in quinoa is also supported by fluorescence chlorophyll *a* analysis, in which similar levels of Fv/Fm at HN and LN conditions are indicating that these plants did not suffer damage of PSII. Furthermore, due the changes in the photochemical (qL) and non- photochemical quenching (NPQ) at LN, regarding HN conditions, it is suggested that both landraces experienced dynamic regulations in the use of light, modifying the energy used on photochemical process and/or in the energy dissipated to the environment as heat. The major difference in these aboveground measured parameters between landraces was the capacity of Socaire plants to maintain qL values under LN conditions. Considering that photosynthesis is the principal process that explains qL values in Quinoa [28], this response could be the basis of the different amount of leaf protein between landraces under LN conditions.

It is known that LN supply (and other stress conditions) induces production of secondary metabolites, triggering protein degradation and increasing photorespiration [31]. All these processes induce a rise in cellular NH_4_^+^ concentration, which is toxic and must be incorporated into amino acids in a reaction catalyzed by the GS enzyme. We suggest that the increment in GS activity in roots might be related to a maintenance of cellular homeostasis in the whole plant. GS activity changes in Socaire were related to up-regulation of *CqGLN1*; however, such relationship was not observed in Faro plants. It was suggested that transcriptional and also post-translational modifications could be involved in GS increased affinity and activity in different plant species [31, 32].

Nitrate reductase (NR) is the enzyme that catalyze the limiting reaction in the reduction of NO_3_^−^. It is known that NR activity is dependent on NO_3_^−^ supply; therefore, an increased activity of this enzyme at LN conditions was an unexpected observation (Fig. 5). A plausible explanation of this result is that NR in roots is modulated in a different way than in shoots, so root NR could be activated at minor NO_3_^−^ concentrations [33]. This could be an explanation in Socaire plants, but not in Faro plants, which NR transcript levels dropped by half (Suppl. Table S2). Further studies are necessary in order to understand the biochemical and molecular regulation of key enzymes of root N physiology in this species.

### Phylogenetic and gene expression studies of NO_3_^−^ transporters in quinoa

Four gene families of transporters are involved in nitrate uptake in plants. They include the NPF (NITRATE TRANSPORTER 1/PEPTIDE TRANSPORTER FAMILY) transporters (low affinity nitrate transporter system or LATS) and the NRT2 (NITRATE TRANSPORTER 2) family (high affinity nitrate transporter system or HATS) [12]. We have characterized, by phylogenetic analysis, different LATS and HATS gene homologues in quinoa and two other plant species from the Chenopodioideae subfamily (*Spinacia oleracea* and *Beta vulgaris*; Fig. 6).

NPF family includes a large number of genes (up to 53 members in *A. thaliana*). Arabidopsis *NPF6.3 (NTR1.1/CHL1)* was the first nitrate transporter identified. This gene is expressed in several root tissues, where this transporter performs nitrate uptake from soil [13]. *NPF6.3 (NTR1.1/CHL1)* was firstly identified as low affinity nitrate transporter but, later on, it was observed that this transporter can sense the external nitrate concentration and alter their transport activity. Thus, this protein has been proposed as transceptor with N-sensing function [14]. *NPF6.3* can switch to high-affinity activity in LN conditions, and an increase in *NPF6.3* mRNA transcripts in presence of low levels of NO_3_^−^ was observed [11, 13]. In fact, knockout *NPF6.3* mutants are defective in high affinity NO_3_^−^ uptake [34]. We identified up to four homologues of *NPF6.3* in quinoa (Fig. 6a). The presence of these homologues probably results from gene duplication events and suggests a fine-tuning regulation of root N uptake by the members of this gene clade in quinoa. We have studied the expression of one of these *NPF6.3* homologues (*NPF6.3-like*, Acc. no. XM_021912755) in roots of Socaire and Faro landraces submitted to either HN or LN supply (Fig. 7a). At HN conditions, gene expression of *NPF6.3-like* is significantly higher in Faro plants than in Socaire plants, suggesting a role of this gene in low-affinity NO_3_^−^ uptake, and a putative higher activity and/or efficiency for NO_3_^−^ uptake in Faro plants. However, this gene is significantly upregulated in Socaire plants at LN supply, suggesting a high affinity NO_3_^−^ transport function for this gene in this landrace (Fig. 7a). In Arabidopsis, phosphorylation of a Thr residue in LN conditions promotes *NPF6.3* high affinity NO_3_^−^ transport [13]. Interestingly, all four *NPF6.3* homolog sequences identified in quinoa have conserved the corresponding Thr residue. It is also interesting to note that two *NPF6.3* homologues identified in spinach (Fig. 6a) are mainly expressed in roots, they are upregulated at HN conditions, and both homologues presented different expression patterns when comparing low N and high N plant varieties [35]. Thus, functional characterization studies must be performed in order to elucidate the functional roles of these transporter homologues in *C. quinoa* and in other plants of the Chenopodioideae subfamily.

Regarding HATS, seven *NRT2* genes were identified and characterized in *Arabidopsis*, and it is known that the *NRT2* family plays and important role in nitrate acquisition from soil under N limitation conditions [14]. It was observed that the constitutive gene *NRT2.5* of *A. thaliana* plays a role in root nitrate influx [36]. It is interesting to note that plants from the Chenopodioideae subfamily have several (up to 9, in the case of *C. quinoa*) homologues that cluster together with this nitrate transporter of Arabidopsis (Fig. 6b), suggesting an important role of these genes in root nitrate uptake in *C. quinoa* and the other plants of the Chenopodioideae subfamily. These homologues are probably the result of gene duplication events.

By another way, *A. thaliana NRT2.1* is the most studied transporter of the *NRT2* family, and it was characterized as the main component of the HATS for nitrate root uptake under most conditions. This gene displays a very strict tissue-specific transcriptional profile, confined to the outer layers of the root tissues, and knockout mutation of this gene results in the loss of up to 75% of the root nitrate influx [36, 37]. Plants of the Chenopodioideae subfamily have no close orthologues for *NRT2.1*, but they have several homologues that cluster together with a clade containing several inducible HATS expressed in roots of *A. thaliana* (that is, *NRT2.1*, *NRT2.2* and *NRT2.4*) and other two homologues (*NRT2.3* and *NRT2.6*) whose function in plants is little known [12]. We have studied the expression of one of these HATS homologues (*High Affinity Transporter 2.1-like*, Acc. no. XM_021864786) in roots of Socaire and Faro plants submitted to either HN or LN supply (Fig. 7b). This gene is significantly induced in both quinoa landraces at LN supply. This result agrees with the expression patterns of its homologues in *A. thaliana* roots, which display a transient upregulation by N starvation [12]. In the case of quinoa, this upregulation was significantly higher in Socaire than in Faro plants, suggesting a putative higher activity and/or efficiency for nitrate uptake at LN supply in Socaire plants. Interestingly, it was reported that expression of a *S. oleracea NRT2.1*-like homologue (Acc. no. XM_022009342) is higher in roots of a low-nitrate content variety than in roots of a high NO_3_^−^ content variety, suggesting a role in NO_3_^−^ uptake; however, no gene upregulation was observed in LN supply conditions [35], suggesting that the role and regulation of this homologue could be different in different plant species of the Chenopodioideae subfamily. It was reported that *A. thaliana NRT2.1* can be involved in lateral root development [36]; concordantly, we observed that Socaire plants display a higher number of lateral roots, and higher total root length, than Faro plants at LN supply (Fig. 4c, d).

No homologues of the tonoplast-specific *NRT2.7* gene of *A. thaliana* were found in Chenopodioideae. This gene is important for nitrate accumulation in seeds of *A. thaliana* [12, 38]. Future studies must elucidate whether these plants of the Chenopodioideae subfamily have nitrate transporter homologues with specific functions in seeds.

Additional studies must be performed in order to elucidate expression patterns, substrate specificity and functional roles of LATS and HATS homologues of quinoa. These studies will allow a characterization of the contribution of these genes to nitrate accumulation and to identify which homologues are more important in order to cope with infertile soils.

## Conclusions

In conclusion, our results suggest that Socaire and Faro landraces have different strategies to cope with low nutrient acquisition. Socaire seeds were able to germinate faster than Faro, which could be associated to a different metabolite composition of seeds. NO_3_^−^ treatment affected the expression of related genes, modulating root morphology and aerial performance differentially among ecotypes. Considering variation on soil quality and N sources on the different places [39], specific adaptations to NO_3_^−^ uptake between ecotypes could be shaping our results. Thus, our work opens new future questions: how different sources of N can influence their uptake and assimilation in a long widely expanded species as quinoa?

Our findings are important as exploration of different quinoa germplasm ecotypes with different sensitivity to NO_3_^−^ limitation. The identification of valuable traits performed here could be used for screening and selecting N tolerant germplasm for their spread in poor soils.

## Methods

### Seed collection

Two landraces of *Chenopodium quinoa* were used in this work: Socaire from Altiplano (San Pedro de Atacama, Atacama Desert, Chile), and Faro from Central Chile. Socaire seeds were collected in a private field (23°34′S 67°54′W, 3500 m.a.s.l.) with the permission of owner. Socaire community and CEAZA were in accordance to research Socaire quinoa germplasm according the agreement related to the project FIA PYT-2014-0280.

Faro seeds were collected in Vicuña City (in an experimental station of the Banco Base Inia Intihuasi (La Pepa, 30°2′S 70°42′W, 616 m.a.s.l.) with all the permission required by the Institution (http://163.247.128.32/gringlobal/search.aspx). Pedro Leon (Banco Base Inia Intihuasi) performed formal identification of the specimen plants. It is worth to note that *C. quinoa* is not endangered species, and experimental research was in according to the practices of SAG (Servicio Agricola y Ganadero) of Chile.

Seeds of both landraces were collected in summer (February) in each location. Total percent and available Nitrogen were 0.09% and 46 mg/kg in Socaire soils, and 0.12% and 42 mg/kg in Vicuña soils, respectively. Further characterization of agroecological conditions in both places are described in Garcia et al. and Montes et al. [40, 41]. Collected seeds were stored in dry conditions at room temperature for 5 months, until experiments were performed.

### Seed characterization and metabolite profiling

Seeds from three randomly selected plants were collected separately for the different experiments (characterization, metabolomics profile and germination). For weight measurements, Socaire and Faro quinoa grains were dried in an oven at 80 °C overnight, and 1000-seed weight of each landrace was measured. One hundred milligram of grinding seeds was used to quantify N content by the method of Kjeldahl et al. [42].

For the metabolomic analysis, seeds were frozen, ground to powder and lyophilized to generate 50 mg samples for each sample. Metabolite profiling and analysis was performed at the West Coast Metabolomics Center (UC Davis, Davis, CA, USA), according to the methodology described in Botanga et al. [43]. The homogenization, extraction and derivatization of the seed powder was according to Fiehn et al. [44]. Chromatography was performed following the protocol described in Botanga et al. [43]. Metabolites were identified using the Fiehnlib libraries [45]. Data were further processed using the algorithms implemented in the open-source BinBase metabolome database [46].

Raw data were normalized, filtered and analyzed using the MetaboAnalyst 3.0 webserver [47]. Heatmap plots (to visualize metabolites profiles) were performed using the pheatmap R package. Data were scaled by Z-sScore to capture metabolites with similar behavior. Clustering of metabolites was performed using the default pheatmap Ward method. Metabolites pathway information was obtained from KEGG API (https://www.kegg.jp/kegg/rest/keggapi.html) using a Shell script.

### Germination

Fifty seeds of three different individual of both landraces were used for each experiment. Seeds from both *C. quinoa* landraces were placed on Petri dishes with filter paper; sterile distilled water was added as needed to ensure adequate moisture for germination. Seeds were incubated for 48 h at 24 °C in the dark. The number of germinated seedlings was recorded every 4 h for 48 h. This experiment was performed in triplicate. Germination was considered complete when the radicle emerged from the seed. Germination rate (number of germinated seeds/h) was recorded for seeds of each landrace during 48 h [48]. The germination percentage, defined as [number of germinated seeds/number of total seeds] × 100 [49] was also recorded every 4 h up to 48 h after seed imbibition.

### Determination of sufficient and low nitrate supply

In order to determine the sufficient and limiting NO_3_^−^ supply concentrations for both Socaire and Faro plant landraces, germinated seeds presenting similar lengths of emerged radicle were transplanted into 700 mL pots containing sand:perlite (1:1). Seedlings were grown under growth chamber conditions (21 °C, 16 h light/8 h dark photoperiod, 57% relative humidity) for 20 days. Plants were watered with MS 407 nutrient medium, which contained the macro- and micronutrients described by Murashige and Skoog [50], except nitrate, potassium and phosphate. In order to compensate for the lack of those nutrients, nutrient solution was supplemented with 85 mg/L KH_2_PO_4_, 950 mg/L KCl, and different concentrations of NO_3_^−^ as KNO_3_. Six nitrogen treatments (0.0, 0.5, 2.0, 5.0, 20 and 100 mM NO_3_^−^) were applied in order to determine sufficient and limiting nitrate supply conditions for both quinoa genotypes. Ten pots (with 3 plants each) were submitted to each nitrogen treatment.

Seedlings were irrigated with the different solutions at field capacity during the time of the experiment. Plants were also irrigated in order to compensate for water losses during the time of the experiment. At the end of the experiment, samples of whole plants were collected, and growth parameters were measured in order to evaluate sufficient and low nitrogen supply concentrations.

### Seedling performance at sufficient and low nitrate supply

In order to study the performance of both Quinoa landraces at sufficient and limiting N conditions during establishment, seedling of quinoa of both landraces were grown with irrigation at two NO_3_^−^ concentrations, previously established with the experiment mentioned above (Section 2.4): 20 mM and 0.5 mM NO_3_^−^, corresponding to sufficient (HN) and low (LN) NO_3_^−^ supply, respectively. Thirty pots of 700 mL (3 plants per pot) were used for each N supply condition per landrace, using the same plant growth conditions described above (Section 2.4). The experiment was run for 24 days in a completely randomized design, that included additional plants grown in order to prevent bordering effect. At the end of the experiment, plants with at least four true leaves were collected.

For the study of the mechanisms involved in N uptake and assimilation, Chlorophyll a fluorescence, biomass, chlorophylls and protein content, NR and GS enzymatic activities and gene expression, and nitrate transporter gene expression were quantified in plants of both landraces submitted to both N treatments.

### Plant morphometric analyses

Leaf area (*n* = 5 plants of each landrace submitted to each treatment) was measured through image analysis using the ImageJ software. Dry weights of leaves, shoots and roots (*n* = 5 plants of each landrace submitted to each treatment) were determined by drying the tissues at 60 °C for 48 h till constant weight. Number of secondary roots and total root length (*n* = 5 plants of each landrace submitted to each treatment) were determined by image analysis using the WinRhizo software [51].

### Protein content quantification

Leaves and roots (*n* = 5 plants of each landrace submitted to each treatment) were ground in liquid nitrogen and homogenized in 400 μL of extraction buffer (50 mM Tris-HCl pH 7.8, 1 mM EDTA, 1 mM DTT, 10 mM MgSO_4_, 5 mM sodium glutamate, 10% ethylene glycol). After centrifugation (10,000 g for 10 min), the supernatant was used to measure total protein content by the Bradford assay [52]. Bovine serum albumin was used as standard.

### Chlorophyll quantification and chlorophyll fluorescence measurements

Chlorophyll *a* and *b* contents were determined spectrophotometrically following the method described by Lichtenthaler and Buschmann [53].

Chlorophyll (Chl) fluorescence measurements were performed (n = 5 plants of each landrace submitted to each treatment) using a portable fluorometer (FMS 2, Hansatech Instruments Ltd., Norfolk, UK). Leaves were dark-adapted for 20 min prior to measurements. Measurements were performed at chamber temperature. Actinic light used was of 300 μmol photons m^− 2^ s^− 1^, as described in Bascuñán-Godoy et al. [54]. Fluorescence parameters were calculated as described in Maxwell and Johnson and Kramer et al. [55, 56].

### Nitrate reductase and glutamine synthetase activities of roots

Both nitrate reductase (NR; EC 1.6.6.1) and glutamine synthetase (GS; EC 6.3.1.2) enzymes catalyze the limiting steps in the reduction of NO_3_^−^ to NH_4_^+^ (primary assimilation), and the incorporation of NH_4_^+^ into amino acids, respectively. NR activity was measured in roots (*n* = 5 plants of each landrace submitted to each treatment) according to Kaiser and Lewis [57]. Total protein was extracted as described above, and the reaction was started by addition of 150 μL of reaction buffer (50 mM KH_2_PO_4_-KOH buffer, pH 7.5; 10 mM KNO_3_ and 0.1 mM NADH) to 100 μL of soluble protein extract. Samples were incubated at 30 °C for 15 min. The NADH oxidation was measured by spectrophotometrical methods at 340 nm. GS activity was measured in roots (*n* = 5 plants of each landrace submitted to each treatment) by the formation of γ-glutamyl hydroxamate using the method described by O’Neal and Joy [58]. In order to perform the enzymatic reaction, 400 μL of protein extract were mixed with 150 μL of reaction buffer (100 mM Tris-HCl, pH 7.8; 50 mM sodium glutamate, 5 mM hydroxylamine hydrochloride, 50 mM MgSO_4_ and 20 mM ATP) and incubated at 30 °C for 20 min. The reaction was stopped with detection solution (0.37 M FeCl_3_, 0.67 M HCl and 20% trichloroacetic acid). Glutamyl hydroxamate was measured spectrophotometrically at 540 nm using γ-glutamyl hydroxamate as a standard.

### Phylogenetic analysis of nitrate transporters

Nitrate transporter (LATS and HATS) homolog sequences of *C. quinoa* and two other species from the Chenopodioideae subfamily (*Beta vulgaris* and *Spinacia oleracea*) were obtained from the Phytozome (https://phytozome.jgi.doe.gov/pz/portal.html) and NCBI (https://www.ncbi.nlm.nih.gov) databases, using the BLAST algorithm and the *Arabidopsis thaliana* homologues as query. The evolutionary history of these genes was inferred using the Maximum-Likelihood method based on the Tamura-Nei model [59]. Node robustness was assessed by the bootstrap method (N = 1000 pseudoreplicates). Phylogenetic analyses were performed with MEGA7 [60]. After a first characterization by phylogenetic analysis, a LATS and a HATS homologue of quinoa were selected for gene expression studies.

### RNA extraction, first strand cDNA synthesis and quantitative real-time PCR

Total RNA was isolated from quinoa roots using RNeasy Plant Mini kit (Qiagen). The RNA obtained was treated with RNase-free DNase I (Qiagen) and quantified with a NanoDrop 1000 spectrophotometer (Thermo Scientific, USA). RNA intactness was verified by visual inspection of integrity of 28S and 18S rRNA bands in denaturing formaldehyde/agarose gel electrophoresis. RNA was stored at − 80 °C for further use.

RNA (1 μg) was reverse-transcribed into single-stranded cDNA templates using the PrimeScript™ RT reagent Kit (Takara) and oligo-p (dT)_15_ primer. Reverse transcription was done in equiproportions (i.e., from equal quantity of RNA) within all compared samples from each experiment. The cDNA synthesis reaction mixture was diluted 10-fold in distilled water before using in real-time PCR.

Gene-specific primers for real-time PCR reactions were designed using Primer 3 input software v. 0.4.0 (http://bioinfo.ut.ee/primer3-0.4.0/primer3/input.htm), to have melting temperatures of 58–60 °C and generate PCR products of 50–200 bp. The elongation factor (*EF1-alpha*) was used as endogenous (housekeeping) gene in order to normalize experimental results [26]. Primer sequences and the accession numbers of the corresponding genes are as follows: *EF1-alpha* (forward, 5′- GTACGCATGGGTGCTTGACAAACTC-3′; reverse, 5′-TCAGCCTGGGAGGTACCAGTAAT-3′; GenBank accession no. XM_021860126); *Nitrate reductase [NADH]-like* (forward, 5′ -AGGACTGGACCATTGAGGTG-3′, reverse 5′- GCTGCAGAACCCCAATTAAA-3′; Acc. no. XM_021892662.1); *Glutamine synthetase cytosolic isozyme 1–1-like (forward 5′-* AAAGGATATTTCGAGGACAGGAGG-3′, reverse 5′- CTTGAGAGACAGCTGCAGATT-3′); Acc. no. XM_021911887.1; *NRT1/PTR Family* (that is, *NPF*) *6.3-like* (a LATS homologue) (forward, 5′-GAGACATGGCTAGCTGAGGA-3′; reverse, 5′-CCTTTTAGGCATGACATTAGCTACT-3′; Acc. no. XM_021912755); *High-affinity Nitrate Transporter* (that is, *HATS*) *2.1-like* (forward, 5′-ATGTTGCTGAGTACGACGAC-3′; reverse, 5′- GGGACGTTGTGTAGGGGTAG-3′; Acc. no. XM_021864786).

Each PCR reaction contained 10 μL of 2X SYBR Green PCR master mix (Agilent Technologies), 50 ng of cDNA, and 0.45 μM (final concentration) of each primer, in a final volume of 20 μL. Real-time PCR reactions were run in an Agilent Mx3000P QPCR System (Agilent Technologies). The initial denaturing time was 3 min at 95 °C, followed by 35 PCR cycles consisting of 95 °C for 30 s and 60 °C for 20 s. After the PCR cycles, the purity of the PCR products was checked by analysis of the corresponding melting curve. The comparative 2^(−ΔΔCT)^ method was used to quantify the relative abundance of transcripts [61]. Experiments included three biological replicates, and three technical replicates for each biological replicate were performed.

### Statistical analyses

Data were analyzed with the STATISTICA v6.0 software package (Statsoft Inc., Tulsa, OK, USA, www.statsoft.com). One-way ANOVA was used to identify differences between landraces during germination.

To analyze metabolite profiles and compare both landraces, first an exploratory data analysis was developed using principal components analysis (PCA) by means of scatterplot3d R package (Suppl. Fig. S3). PCA analysis revealed that the first principal component (PC1) explains 94% of the variance while PC2 and PC3 account for 4 and 1%, respectively, grouping the data in two main clusters that correspond to Socaire and Faro landraces. Thus, the variation in the PCA components was most likely attributable to variance between landraces while smaller variations in PC2 and PC3 were attributable to differences between replicates.

From the 371 metabolites detected, only 161 with KEGG compound identity were analyzed (Suppl. Table S1). To compare metabolites with pathway information the 100 metabolites with the lowest variance between replicates from each landrace were selected and those without KEGG pathway information assigned were removed (Suppl. Table S1). Thus, 85 remnant metabolites were analyzed by a heat-map, including metabolite clustering analysis with pathway information from KEGG.

For the determination of sufficient and low nitrate supply, we used two-way ANOVA (level of significance *P* < 0.05). Data from effects of nitrate supply on the different quinoa landraces (Socaire and Faro) were analyzed by two-way ANOVA. Fisher test was used to identify means with significant differences (level of significance *P* < 0.05).

## Supplementary information

**Additional file 1: Figure S1.** Seed germination rate in Socaire and Faro landraces. Measurements were performed every 4 h during 24 h (50 seeds per plate, *n* = 3). Asterisks indicate significant differences between landraces.

**Additional file 2: Figure S2.** Effect of N deficiency on growth parameters in *C. quinoa* plants. Plants subjected to differing NO_3_^−^ supplies from 0 to 100 mM per 20 days. (a) Images of seedlings after 20 days of treatment (b) Leaf area and (c) Shoot fresh weight. Values are means ± SE (*n* = 7). Different letters show statistical differences using two-way ANOVA considering landraces and nutrition treatment (20 mM and 0.5 mM NO_3_^−^) as factors (Fisher LSD test; *P* < 0.05).

**Additional file 3: Figure S3:** Principal component analysis (PCA) of metabolites from seeds of Socaire and Faro landraces.

**Additional file 4: Table S1.** Raw metabolomics data used for heatmap and metabolite clustering analysis.

**Additional file 5: Table S2.** Data of physiological. Biochemical and molecular parameters measured in Socaire and Faro quinoa plants submitted to HN (20 mM NO_3_^−^) and LN (0.5 mM of NO_3_^−^) supply, and depicted in flow chart (Fig. 5). Values are mean ± SE (*n* = 5). Two-way ANOVA was used to denote significant differences considering landraces and nitrogen treatment as factors (Fisher LSD test; *P* < 0.05). Significant *P*-values are in red.

## Data Availability

All data generated during this study are included in this published article and its supplementary information files.

## Abbreviations

Chl: Chloropyll
Fv/Fm: Maximal efficiency of PSII
GOGAT: Glutamine Oxoglutarate Aminotransferase
GS: Glutamine Synthetase
HATS: High Affinity Transport Systems
HN: Sufficient nitrate
KNO3: Saltpeter
LATS: Low Affinity Transport Systems
LN: Low nitrate
MCL: Maximum Composite Likelihood
N: Nitrogen
NO_3_^−^: Nitrate
NiR: Nitrite Reductase
NPQ: Non-photochemical quenching
NR: Nitrate Reductase
PSII: Photosystem II
qL: Photochemical quenching
