# Supplementary figures and images for: Seed characterization and early nitrogen metabolism performance of seedlings from Altiplano and coastal ecotypes of Quinoa
Source: BMC Plant Biol. 2020 Jul 21;20:343. doi: 10.1186/s12870-020-02542-w (PMC7372889; doi:10.1186/s12870-020-02542-w)

## Slide 1
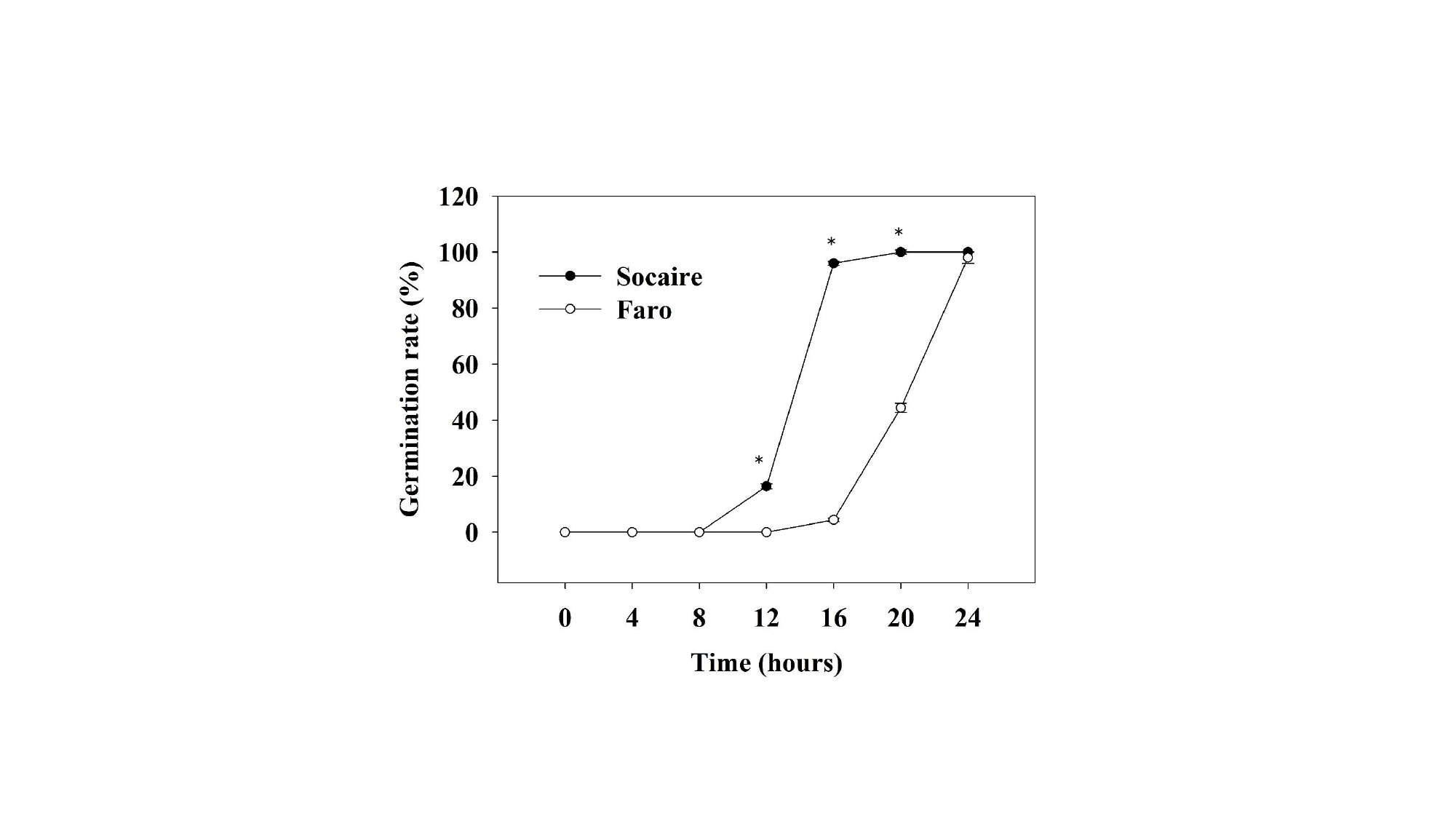

*
*
*

Supplement: Supplementary file 1 — Additional file 1: Figure S1. Seed germination rate in Socaire and Faro landraces. Measurements were performed every 4 h during 24 h (50 seeds per plate, n = 3). Asterisks indicate significant differences between landraces. [file 12870_2020_2542_MOESM1_ESM.pptx]

## Slide 1
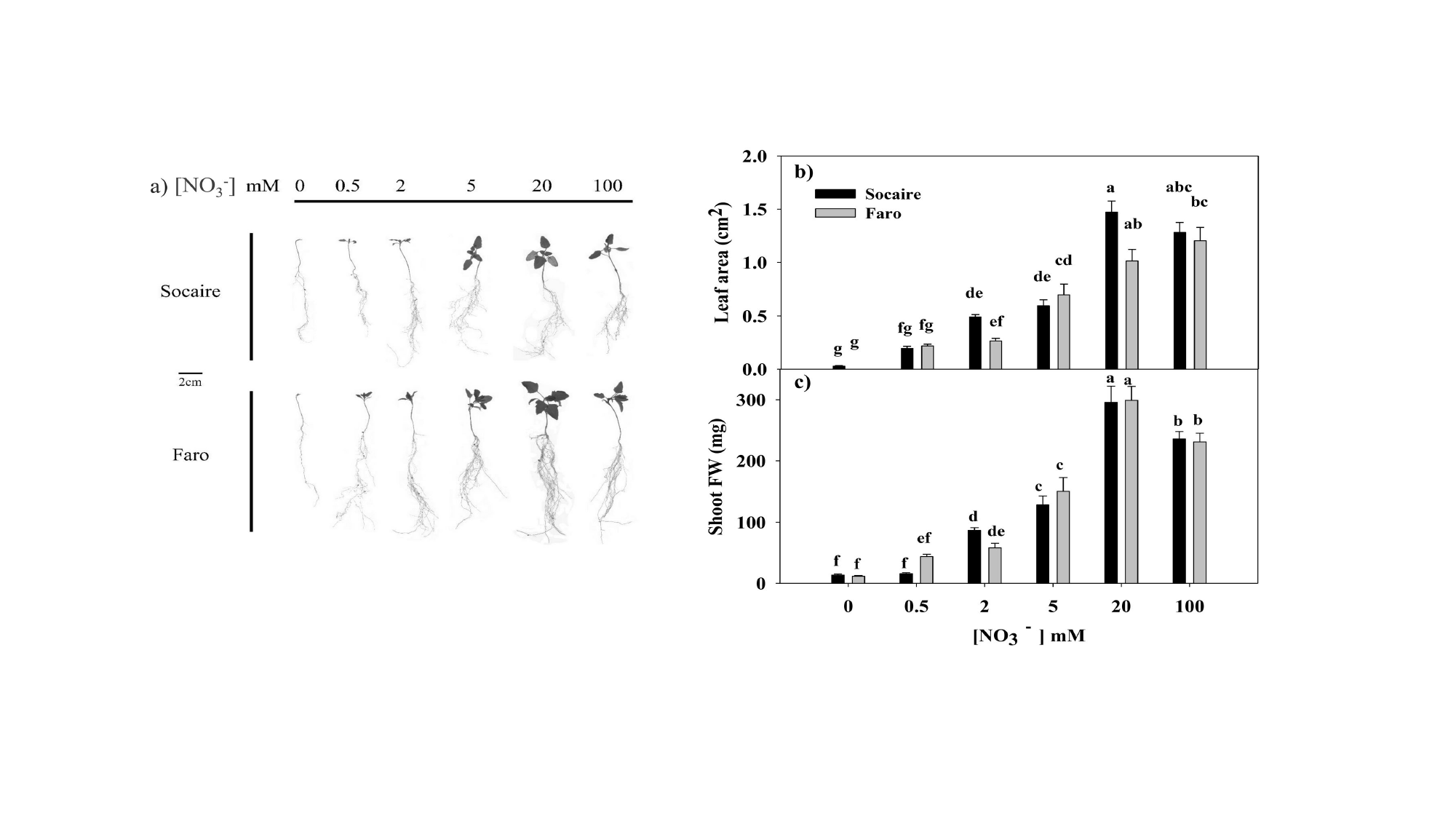

Supplement: Supplementary file 2 — Additional file 2: Figure S2. Effect of N deficiency on growth parameters in C. quinoa plants. Plants subjected to differing NO3− supplies from 0 to 100 mM per 20 days. (a) Images of seedlings after 20 days of treatment (b) Leaf area and (c) Shoot fresh weight. Values are means ± SE (n = 7). Different letters show statistical differences using two-way ANOVA considering landraces and nutrition treatment (20 mM and 0.5 mM NO3−) as factors (Fisher LSD test; P < 0.05). [file 12870_2020_2542_MOESM2_ESM.pptx]

## Slide 1
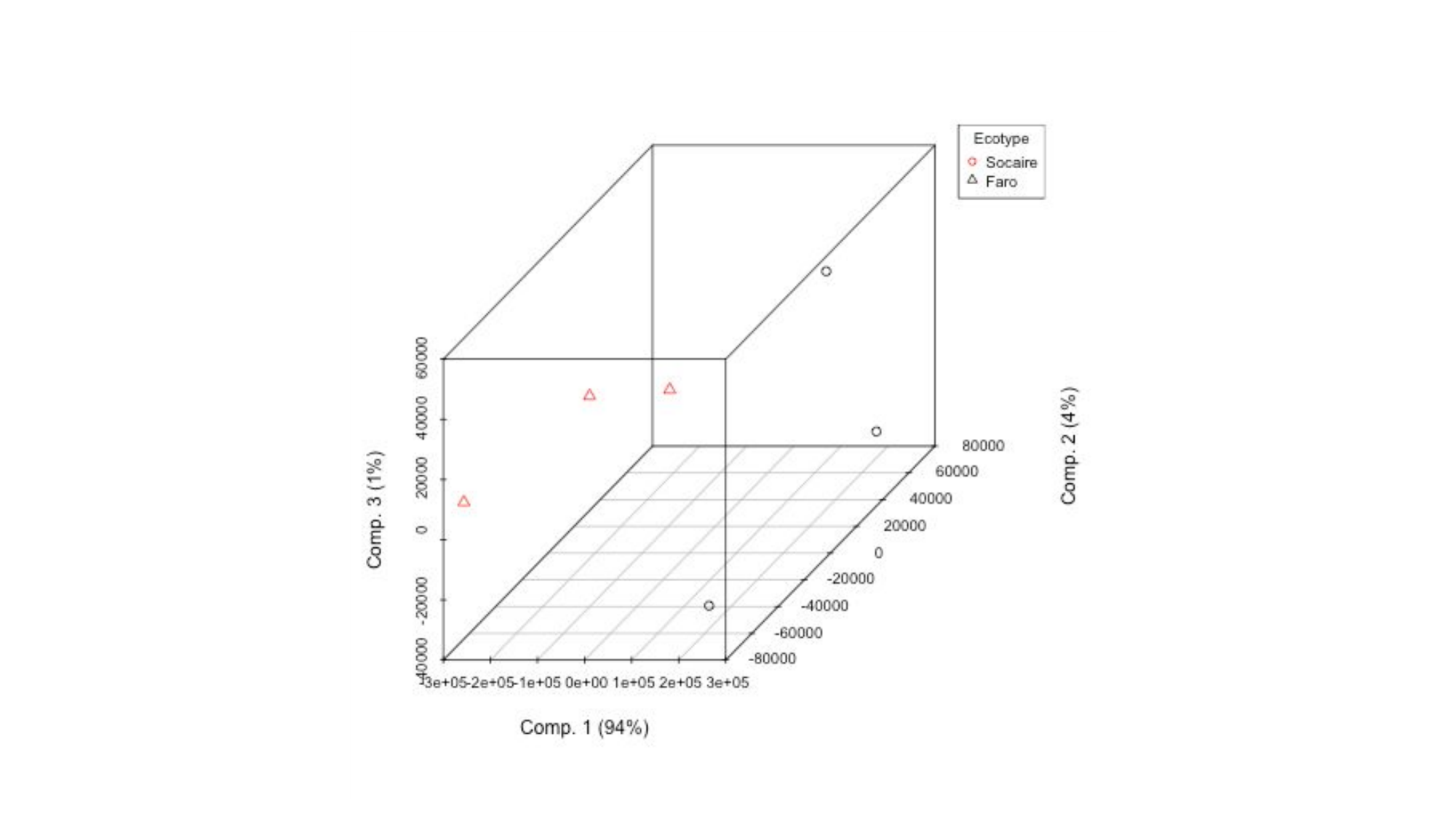

Supplement: Supplementary file 3 — Additional file 3: Figure S3: Principal component analysis (PCA) of metabolites from seeds of Socaire and Faro landraces. [file 12870_2020_2542_MOESM3_ESM.pptx]
