# Supplementary material for: Seed characterization and early nitrogen metabolism performance of seedlings from Altiplano and coastal ecotypes of Quinoa
Source: BMC Plant Biol. 2020 Jul 21;20:343. doi: 10.1186/s12870-020-02542-w (PMC7372889; doi:10.1186/s12870-020-02542-w)
Supplement: Supplementary file 5 — Additional file 5: Table S2. Data of physiological. Biochemical and molecular parameters measured in Socaire and Faro quinoa plants submitted to HN (20 mM NO3−) and LN (0.5 mM of NO3−) supply, and depicted in flow chart (Fig. 5). Values are mean ± SE (n = 5). Two-way ANOVA was used to denote significant differences considering landraces and nitrogen treatment as factors (Fisher LSD test; P < 0.05). Significant P-values are in red. [file 12870_2020_2542_MOESM5_ESM.docx]

|  | Socaire | | | | |  |  |  |  | Faro | | | | | | |  | ¤ | * | x |
| --- | --- | --- | --- | --- | --- | --- | --- | --- | --- | --- | --- | --- | --- | --- | --- | --- | --- | --- | --- | --- |
|  | HN | | |  | LN | | |  |  | HN | | |  | LN | | |  | Landraces | N | L x N |
| Leaf protein (mg FW^-1^) | 11.0 | ± | 1.8 |  | 12.3 | ± | 2.2 |  |  | 15.7 | ± | 1.6 |  | 5.5 | ± | 0.3 |  | 0.52 | 0.03 | 0.01 |
| Root protein (mg FW^-1^) | 1.00 | ± | 0.12 |  | 1.33 | ± | 0.21 |  |  | 1.02 | ± | 0.06 |  | 0.98 | ± | 0.05 |  | 0.22 | 0.29 | 0.19 |
| Chl *a (*µg FW^-1^*)* | 82.8 | ± | 7.6 |  | 34.7 | ± | 2.9 |  |  | 95.0 | ± | 16.3 |  | 23.1 | ± | 1.7 |  | 0.97 | <0.001 | 0.22 |
| Chl *b* (µg FW^-1^) | 26.6 | ± | 3.5 |  | 10.0 | ± | 0.7 |  |  | 26.4 | ± | 4.2 |  | 7.0 | ± | 0.5 |  | 0.58 | <0.001 | 0.62 |
| Chl *a* /Chl *b* | 3.2 | ± | 0.04 |  | 3.5 | ± | 0.02 |  |  | 3.6 | ± | 0.02 |  | 3.3 | ± | 0.023 |  | 0.16 | 0.91 | 0.01 |
| Fv/Fm | 0.76 | ± | 0.02 |  | 0.8 | ± | 0.02 |  |  | 0.8 | ± | 0.01 |  | 0.8 | ± | 0.004 |  | 0.33 | 0.44 | 0.27 |
| NPQ | 0.22 | ± | 0.03 |  | 0.6 | ± | 0.11 |  |  | 0.3 | ± | 0.11 |  | 0.5 | ± | 0.305 |  | 0.96 | 0.07 | 0.74 |
| qL | 0.60 | ± | 0.07 |  | 0.6 | ± | 0.06 |  |  | 0.8 | ± | 0.03 |  | 0.5 | ± | 0.121 |  | 0.88 | 0.20 | 0.06 |
| Root NR activity  (nmol NO_2_^-^ mg^-1^ h^-1^) | 46 | ± | 6 |  | 119 | ± | 62 |  |  | 47 | ± | 9 |  | 132 | ± | 16 |  | 0.84 | 0.04 | 0.86 |
| Root GS activity  (nmol Glu mg^-1^ h^-1^) | 5.6 | ± | 0.4 |  | 9.2 | ± | 0.7 |  |  | 3.4 | ± | 0.2 |  | 10.0 | ± | 3.4 |  | 0.71 | 0.02 | 0.43 |
| Root *CqNR* expression (fold change) | 1.0 | ± | 0.0 |  | 5.5 | ± | 0.7 |  |  | 2.6 | ± | 0.3 |  | 1.0 | ± | 0.1 |  | 0.01 | 0.00 | <0.001 |
| Root *CqGLN* expression (fold change) | 1.01 | ± | 0.13 |  | 1.84 | ± | 0.55 |  |  | 1.36 | ± | 0.23 |  | 1.549 | ± | 0.04 |  | 0.92 | 0.13 | 0.32 |
